# Supplementary figures and images for: One Health surveillance of colistin-resistant Enterobacterales in Belgium and the Netherlands between 2017 and 2019
Source: PLoS One. 2024 Feb 23;19(2):e0298096. doi: 10.1371/journal.pone.0298096 (PMC10890735; doi:10.1371/journal.pone.0298096)

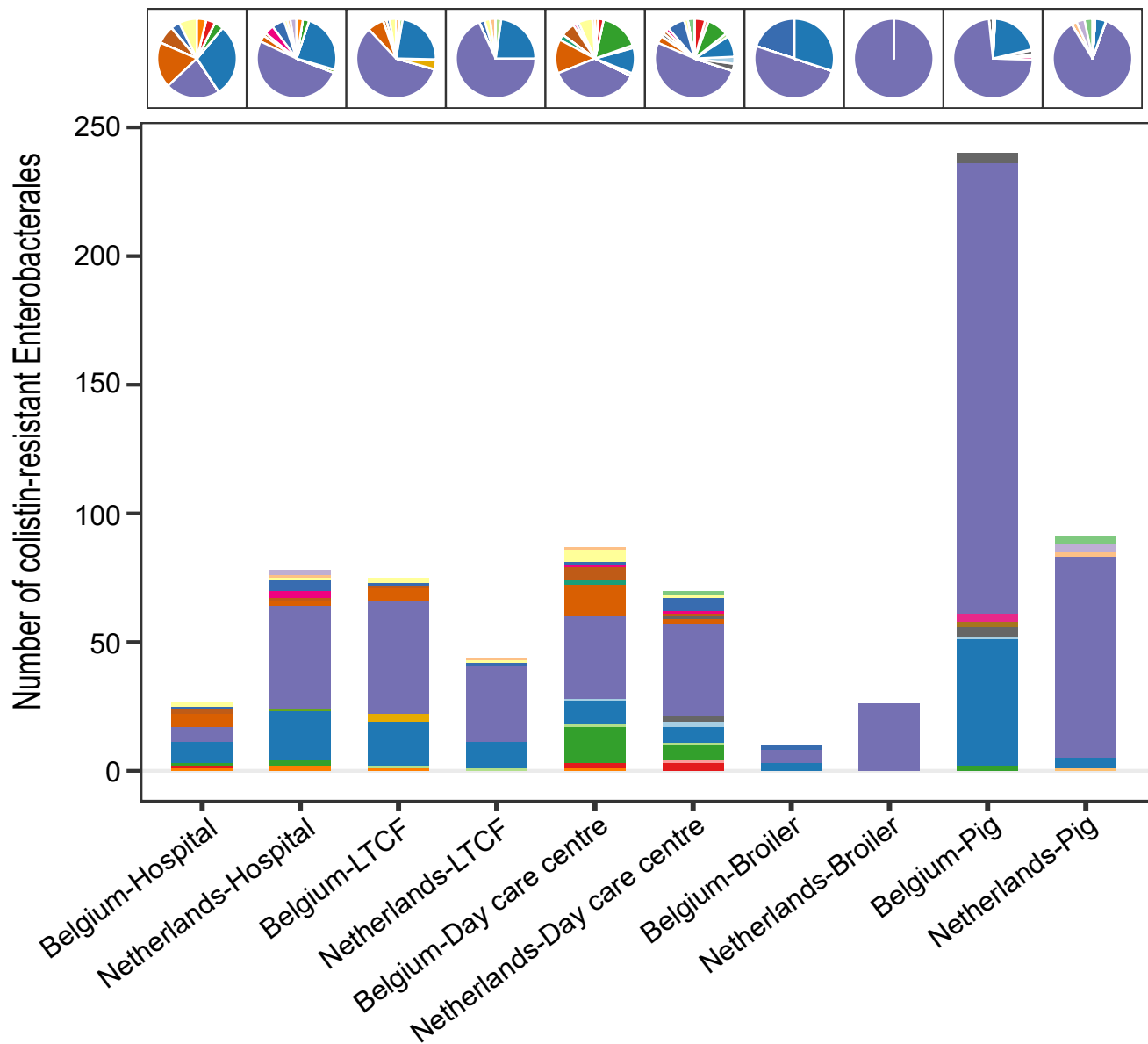

### Species

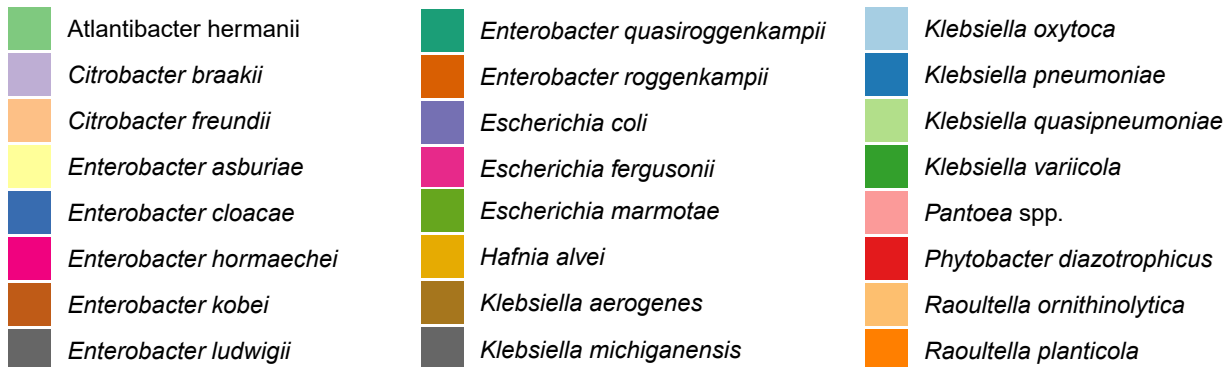

Supplement: S1 Fig — (PDF) [file pone.0298096.s001.pdf]

**A** *Escherichia coli*

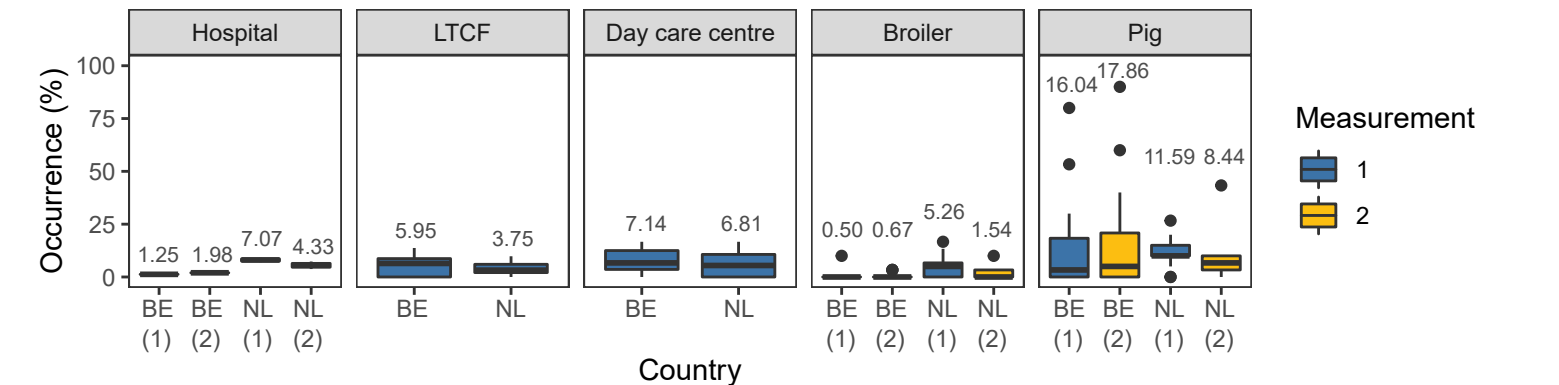

**B** *Klebsiella* spp.

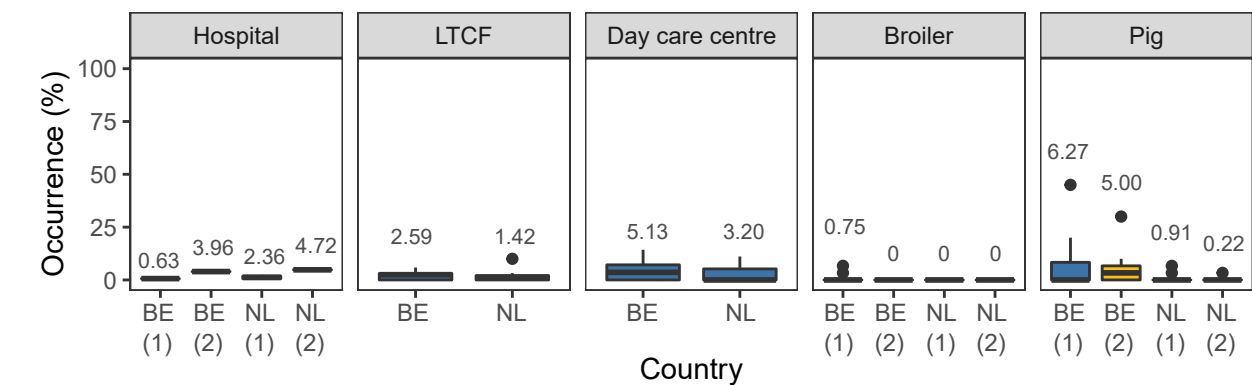

**C** *Enterobacter* spp.

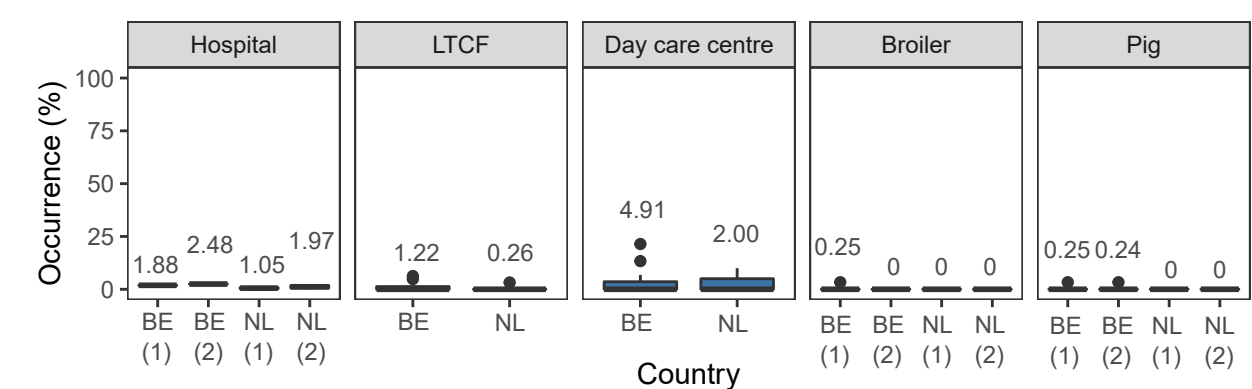

**D** Multidrug-resistant

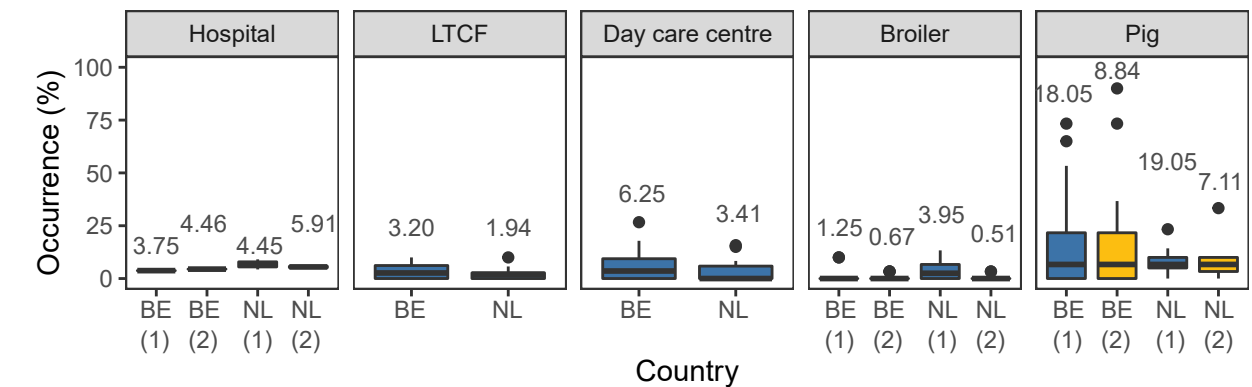

Supplement: S2 Fig — Carriage of colistin-resistant Escherichia coli (A), Klebsiella spp. (B), Enterobacter spp. (C) and multi-drug resistant isolates (D) by humans and animals. The numbers indicated with the boxplots represent the total percentage of positive samples by country, measurement and sector. BE: Belgium, NL: the Netherlands, LTCF: long-term care facility. (PDF) [file pone.0298096.s002.pdf]

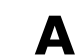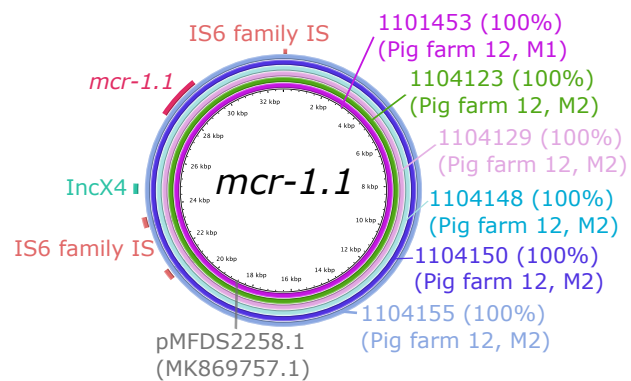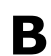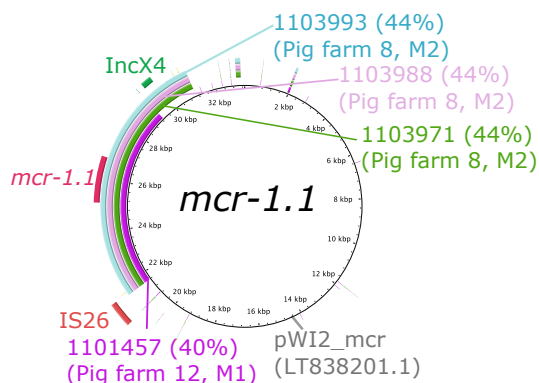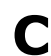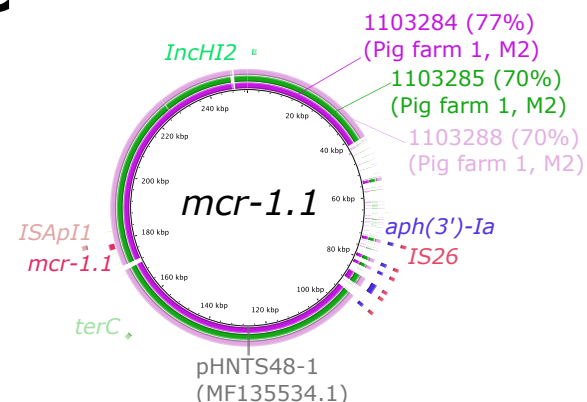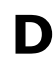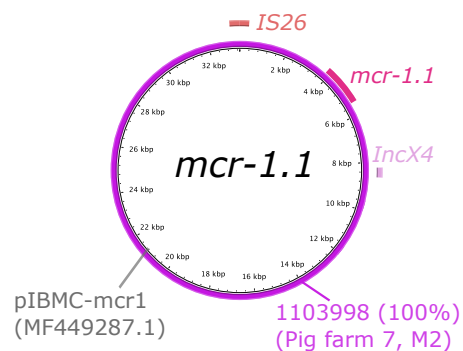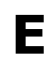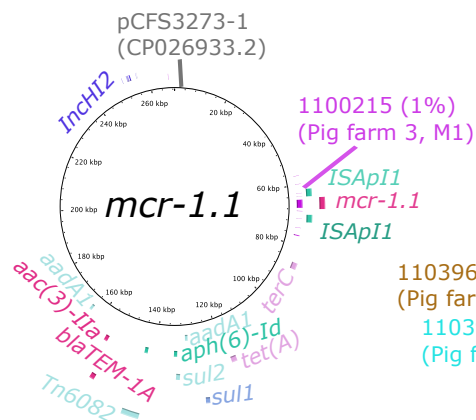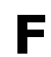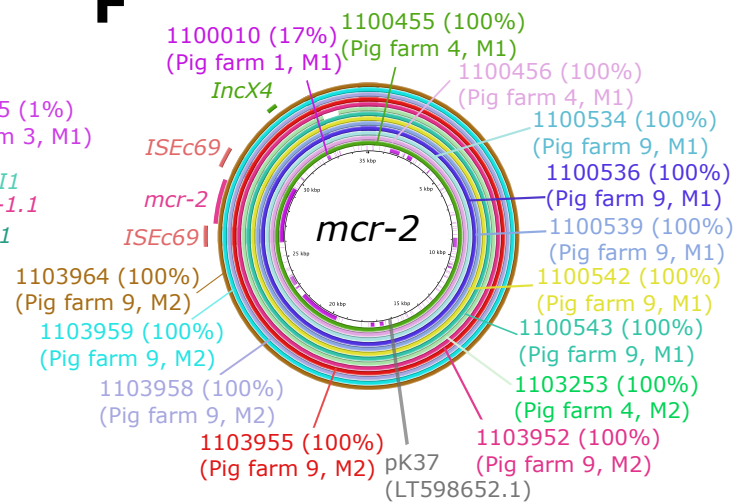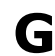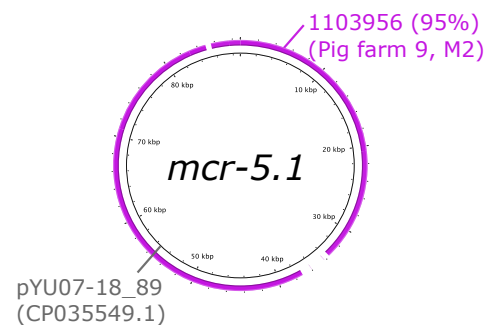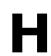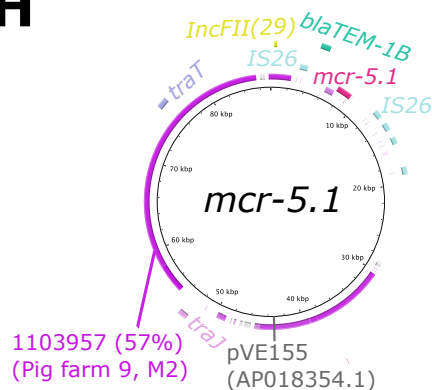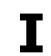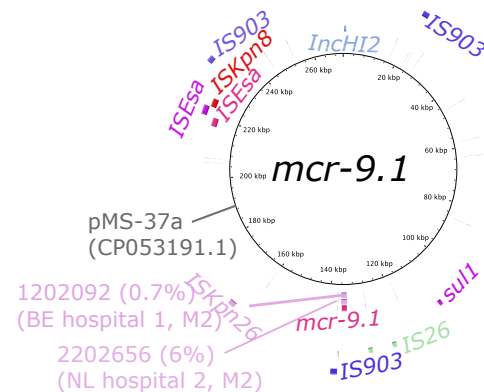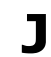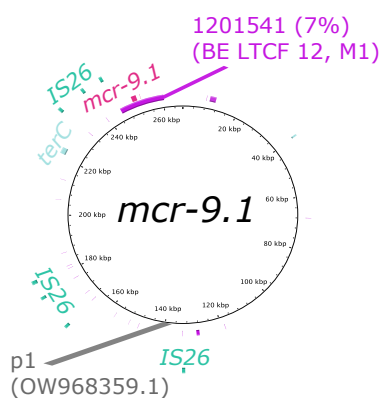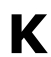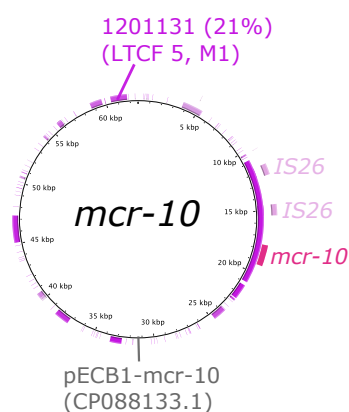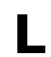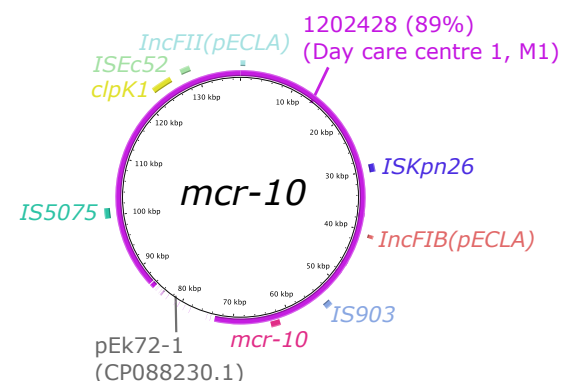

Supplement: S3 Fig — Figure was generated using the BLAST Ring Image Generator (BRIG). Percentages indicate the query coverage of the mcr-containing sequence with the reference plasmid. Isolate ID, origin, insertion sequences, plasmid Inc type, resistance and virulence genes are indicated in different colors. (PDF) [file pone.0298096.s003.pdf]

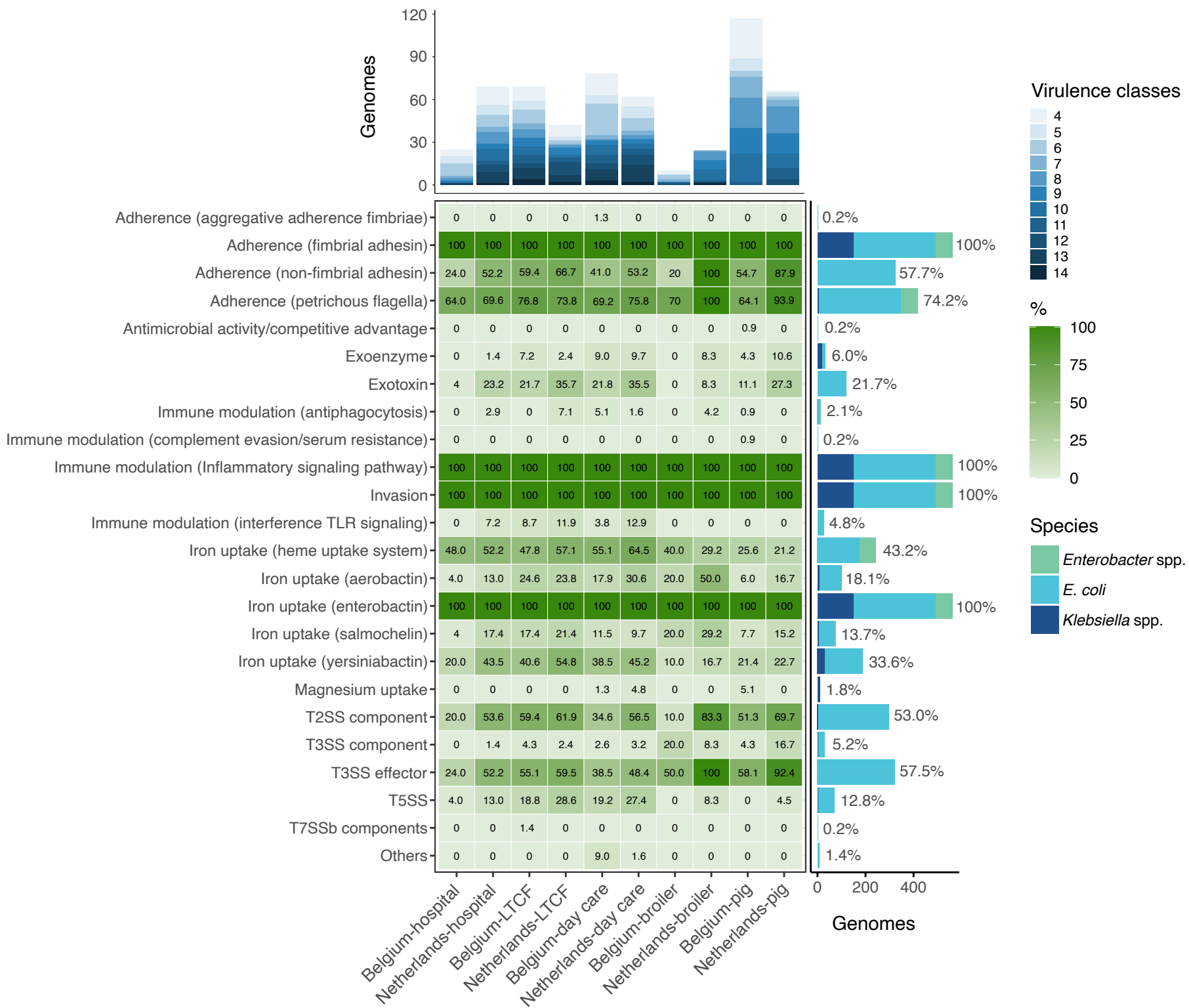

Supplement: S4 Fig — Heatmap of the percentage of ColR-E harboring virulence genes related to virulence classes (y-axis) per One Health sector in Belgium and the Netherlands (x-axis). Barplots show the number of genomes colored by species per virulence class (right) and colored by the number of virulence class per One Health sector (top). LTCF: long-term care facility. (PDF) [file pone.0298096.s004.pdf]
